# A frameshift in time

**DOI:** 10.7554/eLife.78373

**Published:** 2022-04-11

**Authors:** Martina M Yordanova, Pavel V Baranov

**Affiliations:** 1 School of Biochemistry and Cell Biology, University College Cork Cork Ireland

**Keywords:** arterivirus, porcine reproductive, respiratory syndrome virus, PRRSV, ribosome profiling, programmed ribosomal frameshifting, subgenomic mRNAs, Viruses

## Abstract

The efficiency with which ribosomes shift reading frames when decoding viral RNA may change over the course of an infection.

**Related research article** Cook GM, Brown K, Shang P, Li Y, Soday L, Dinan AM, Tumescheit C, Mockett APA, Fang Y, Firth AE, Brierley I. 2022. Ribosome profiling of porcine reproductive and respiratory syndrome virus reveals novel features of viral gene expression. *eLife* **11**:e75668. doi: 10.7554/eLife.75668.

When an RNA virus infects a cell, ribosomes inside the cell decode the genetic information in the virus’s RNA to produce proteins, which are then used to make more viral particles. A single-stranded RNA molecule consists of a sequence of nucleotides that the ribosome reads three at a time. Each triplet, or codon, codes for either an amino acid (the building blocks that form proteins), or signals for the ribosome to start or stop reading the RNA sequence. Therefore, each nucleotide sequence can therefore be ‘read’ by ribosomes in three different ways, or ‘reading frames’, depending on which nucleotide the ribosome starts reading from. Additionally, an ‘open reading frame’ or ORF is a sequence of nucleotide triplets that code for amino acids located between two stop codons in the same reading frame.

Almost all cellular proteins are encoded in a single reading frame, with only rare exceptions (Baranov et al., 2015). Viruses, however, often break this rule in a process termed ‘programmed ribosomal frameshifting’ (Firth and Brierley, 2012; Atkins et al., 2016). This mechanism occurs at specific locations in the nucleotide sequence called frameshift sites, where a proportion of the ribosomes translating the RNA will shift back or forward one nucleotide and start decoding a different reading frame. Meanwhile, the rest of the ribosomes continue reading the original frame. Thus, the same segment of an RNA molecule can be read to produce two protein molecules with distinct amino acid sequences simultaneously.

It is unclear exactly why viruses employ programmed ribosomal frameshifting. One suggestion is that this mechanism allows for a more compact organization of genetic material. Another is that frameshifting could be used for setting a specific ratio between different viral proteins. Most commonly, ribosomal frameshifting occurs during the synthesis of viral polyproteins, long amino acid chains that are processed into smaller proteins with distinct functions. The advantage of organizing protein synthesis in this way is that only one RNA molecule is needed to encode multiple proteins. However, if all these proteins were synthesized as a part of a single polyprotein, they would occur strictly in a one-to-one ratio after being processed. This would be wasteful, since these proteins are needed in different quantities.

So how could the optimal proportions of these proteins be achieved? The low efficiency frameshifting mechanism solves the problem. Proteins that the virus needs in large quantities are encoded early in the sequence in an open reading frame herein referred to as *ORF1A*, while proteins that the virus requires in lower quantities are encoded in a different but overlapping downstream reading frame, herein referred to as *ORF1B* (Figure 1). *ORF1A* is decoded according to standard rules, producing a shorter version of the viral polyprotein. *ORF1B*, on the other hand, is only read by the ribosomes that change reading frame at the frameshift site between *ORF1A* and *ORF1B*, resulting in a longer polyprotein.

**Figure 1.**
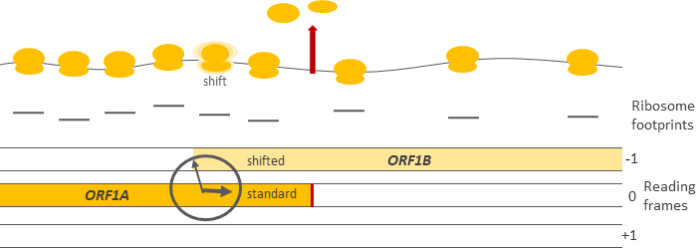
Schematic representation showing how RNA is decoded in the vicinity of the frameshift site between two open reading frames, *ORF1A* and *ORF1B*. Top: most ribosomes (yellow) decoding *ORF1A* terminate at the stop codon (red arrow), release the protein (not shown) and dissociate from the RNA (gray curve). A small proportion of ribosomes, however, shift frames to decode *ORF1B*. The ribosome at the frameshift site is outlined with a fuzzy cloud. Center: the density of ribosome footprints (the lines under each of the ribosomes) revealed by ribosome profiling maps to the positions occupied by ribosomes on the RNA molecule. The ratio between the ribosome footprint density at *ORF1A* and at *ORF1B* can be used as a measure of frameshifting efficiency. Bottom: schematic of the three possible reading frames in a molecule of RNA, each represented by a bar and denoted by –1, 0, and +1. The clock-like nature of the frameshift site drawing alludes to the temporal regulation of frameshifting as revealed by Cook et al.

This type of frameshifting is sometimes referred to as canonical due to its common occurrence in RNA viruses. It was originally assumed that the ratio of products generated from *ORF1A* and *ORF1B* was fixed throughout the virus’s time in the cell (Jacks and Varmus, 1985). Now, in eLife, Ian Brierley, Andrew Firth, Ying Fang and colleagues – including Georgia Cook (University of Cambridge) as first author – report evidence suggesting that this ratio changes over the course of infection (Cook et al., 2022).

The team (who are based at various institutes in the United Kingdom, the United States and China) studied how viral gene expression changes during porcine reproductive and respiratory syndrome virus (PRRSV) infection. To do this, Cook et al. used a technique called ribosome profiling to map which parts of the virus’s RNA sequence were being translated by ribosomes at any given time (Ingolia et al., 2009). These mappings, called ribosome footprints, revealed several new ORFs that encoded previously uncharacterized viral protein products.

Ribosome profiling can also be used to compare how efficiently different proteins are synthesized. For example, in the PRRSV genome the density of footprints mapped to *ORF1A* is higher than the footprint density at *ORF1B*. This happens because only a small proportion of the ribosomes reading *ORF1A* shift reading frame and proceed to *ORF1B* (Figure 1). By calculating the ratio of footprint densities between the two open reading frames it is possible to estimate frameshifting efficiency.

The PRRSV genome is known to contain two frameshift sites: the canonical site between *ORF1A* and *ORF1B*, which is used by many viruses, and a second, rarer frameshift site in *ORF1A* that results in the production of a shorter polyprotein. The genome of a related virus, called the encephalomyocarditis virus, has been shown to have a similar secondary frameshift site that is stimulated by a viral protein (Napthine et al., 2017). The concentration of this viral protein was found to increase over the course of an infection and cause more ribosomes to shift to the other reading frame. However, by measuring the efficiency of both frameshifting sites in PRRSV, Cook et al. showed that this temporal change is not limited to the protein-stimulated frameshifting, but also occurs in the canonical site between *ORF1A* and *ORF1B*.

This finding challenges the current paradigm that regards the canonical frameshifting between *ORF1A* and *ORF1B* as a mechanism that enables a fixed ratio between polyprotein products. The temporal change detected in PRRSV suggests that the efficiency of frameshifting may also be altered in other viruses over time. If so, it would be interesting to determine what factors mediate the regulation of the frameshifting event between *ORF1A* and *ORF1B*.

An open question that remains is how changes in frameshifting efficiency along the course of an infection relate to the virus’s virulence and transmissibility. It is possible that changes in efficiency are simply due to alterations in the infected cell that make ribosomes more prone to shifting to another reading frame. However, it may be that regulating the efficiency of frameshifting is beneficial for the virus. Alternatively, the antiviral response of the host may induce frameshifting to alter the ratio of viral proteins and negatively impact the virus. Indeed, it has been previously reported that the formation of viral particles can be disrupted by altering frameshifting efficiency (Dulude et al., 2006).

Whatever the case, the search for cellular factors responsible for changes in frameshifting has already begun (Riegger and Caliskan, 2022). The identification of these factors will provide researchers with new targets for modulating frameshifting efficiency in viruses, potentially revealing new ways to fight off viral infections.
